# Visual performance, safety and patient satisfaction after bilateral implantation of a trifocal intraocular lens in presbyopic patients without cataract

**DOI:** 10.1186/s12886-022-02556-y

**Published:** 2022-08-10

**Authors:** Joaquín Fernández, José F. Alfonso Sánchez, Mark Nieradzik, Beatriz Valcárcel, Noemí Burguera, Alexander Kapp

**Affiliations:** 1 Qvision, Department of Ophthalmology of VITHAS Almería Hospital, 04120 Almería, Spain; 2 Fernández-Vega Ophthalmological Institute, Av. Doctores Fernández Vega, 34, 33012 Oviedo, Asturias Spain; 3 Augenzentrum Michelfeld, Daimlerstraße 60, 74545 Michelfeld, Germany

**Keywords:** Presbyopia, Refractive lens exchange, Trifocal, Multifocal Intraocular Lenses, Visual function, Patient satisfaction

## Abstract

**Background:**

The aim was to evaluate the safety and efficacy of a trifocal intraocular lens (IOL) for the correction of presbyopia and to assess patient satisfaction.

**Methods:**

Records from three centres were reviewed to select presbyopic patients having undergone bilateral refractive lens exchange and implantation of the AT LISA tri 839MP multifocal IOL. Postoperatively, monocular and binocular distance, intermediate and near visual acuities, corrected and uncorrected, and subjective refraction were measured. Patients also completed a quality of life questionnaire. Safety evaluation included IOL stability and postoperative complications.

**Results:**

72 eyes (36 patients) were analysed. No clinically significant difference between pre- and postoperative corrected distance visual acuity (CDVA) was found for monocular or binocular measurements. Mean postoperative monocular CDVA was 0.02 ± 0.04 logMAR. Mean refractive values all improved statistically significantly compared with preoperative baseline (p ≤ 0.0064). Overall, 82.4% of eyes had spherical equivalent within ± 0.5 D and 97.1% within ± 1.0 D of emmetropia with a mean accuracy of -0.10 ± 0.41 D. Spectacle independence for distance, intermediate and near visual acuity was 87.5%, 84.4% and 78.1% respectively, and 78.1% of patients were satisfied with their postoperative, spectacle-free vision. Eight eyes received Nd:YAG laser treatment. No other IOL-related safety issues were reported.

**Conclusion:**

AT LISA tri 839MP multifocal IOL bilaterally implanted in presbyopic patients provided excellent distance, intermediate and near visual outcomes with very accurate correction of refraction. These results were associated with a high level of spectacle independence and patient satisfaction.

**Trial registration:**

Trial registered on https://clinicaltrials.gov/ under the identification NCT03790592 (31/12/2018).

## Background

Presbyopia is a condition that causes deterioration in near vision with ageing and is particularly common in adults over 40 years of age [1]. It appears to be due in part to a change in the viscoelastic properties of the crystalline lens leading to stiffening and a decrease in the amplitude of accommodation [2]. It is a normal, physiological, but irreversible process that affected an estimated 1.8 billion people worldwide in 2015 and is predicted to affect 2.1 billion in 2030 [3].

A number of treatment options are available to achieve presbyopia correction although none has emerged as the optimum solution. Non-surgical treatments include corrective glasses and contact lenses [4], while surgical treatments include corneal procedures such as laser refractive correction or conductive keratoplasty, scleral procedures, phakic intraocular lens implantation, and clear lens extraction followed by IOL implantation [5]. Recently, an increasingly diverse range of IOL types has become available to physicians to correct presbyopia; from the induction of monovision with monofocal IOLs to the implantation of multifocal, extended depth of focus or accommodative IOLs [6]. Refractive lens exchange with multifocal and more specifically trifocal IOL implantation is increasing for presbyopia correction [7]. Trifocal lenses offer the possibility to correct refraction errors while giving the patients a full range of vision from near to far distances and thus may fulfil the desire for postoperative spectacle independence [8–10]. However, trifocal IOLs also have trade-offs inherent to their optical design. Implanted patients can experience optical disturbances such as glare and halos, loss of contrast and reduced quality of mesopic vision [11].

The AT LISA tri 839MP (Carl Zeiss Meditec, Jena, Germany) is a trifocal IOL intended for micro-surgical implantation through ≥ 1.8 mm incisions. Since 2014, nearly 60 publications have repeatedly demonstrated the safety and performance of the lens in cataract patients, see for example [8, 12–16]. Mixed populations of cataract and presbyopic patients with or without cataract, have also been evaluated and all reports show satisfactory outcomes [17–20]. While there is evidence that the AT LISA tri 839MP IOL is a safe and efficient device to correct presbyopia, demonstration in a clinical trial specifically designed for this purpose is absent. The purpose of this study was therefore to evaluate the safety and the performance of the LISA tri 839MP IOL in bilaterally implanted presbyopic patients. The primary objective was to compare postoperative to preoperative monocular CDVA.

## Methods

### Patient population

This multicentre, non-comparative, cohort study was conducted at three sites in Germany and Spain between May 2019 and December 2019. Patients who had already been bilaterally implanted with the AT LISA tri 839MP were contacted and invited to attend a postoperative visit 4 to 12 months after their second surgery. The protocol was developed and approved before the postoperative visits took place. Pre- and intraoperative data were collected retrospectively and postoperative data were collected prospectively.

The protocol was reviewed and approved by the Ethics Committee of each site, followed the tenets of the Declaration of Helsinki and fully complied with the International Conference on Harmonization and Good Clinical Practice guidelines. All patients provided written informed consent prior to enrolment. The trial was registered before the study began with ClinicalTrials.gov, number NCT03790592 (31/12/2018).

The study included patients who had previously undergone bilateral presbyopic lens exchange and in-the-bag implantation of an AT LISA tri 839MP IOL in each eye (retrospective patient selection). Patients were defined as presbyopic if their near vision prescription was + 0.5 D or greater and the patient required reading glasses. This is consistent with the widely accepted definition of presbyopia as: unaided near vision worse than N6 or N8 at 40 cm or preferred reading distance. The main exclusion criteria were the following: preoperative monocular and binocular CDVA worse than 0.2 logMAR, presence of significant posterior capsule opacification, ocular disorder potentially impacting visual acuity and patients having received refractive laser treatment before or during the follow-up. The selected patients were invited to attend a postoperative visit 4 to 12 months after the implantation in the second eye (prospective data collection) for a full ophthalmologic examination and to complete a questionnaire.

#### Surgical technique

Surgeries were performed by three experienced surgeons JF, JA, AK. Standard hydrodissection and cataract extraction by phacoemulsification was performed in all cases. The AT LISA tri 839MP IOL was subsequently implanted in the capsular bag using a qualified injector (BLUEMIXS 180, Carl Zeiss Meditec, Jena, Germany) in combination with a viscoelastic device. At the end of the surgery, any residual ophthalmic viscoelastic device was thoroughly removed by irrigation/aspiration, and side ports and main incision were sealed by hydration. Postoperative treatment and medication were given according to the routine procedure in each centre.

#### Study lens

The AT LISA tri 839MP is a trifocal (+ 3.33 D; + 1.66 D), aspheric, aberration correcting IOL made of hydrophilic acrylic with hydrophobic surface properties. It features a 4-haptics, non-angulated platform. Its overall diameter is 11.00 mm and its optic diameter 6.00 mm. The manufacturer’s A-constant is 118.6.

#### Assessments

Preoperative and surgery-related data was collected retrospectively. Preoperative data collection included medical history, relevant concomitant pathologies and treatments, monocular and binocular, uncorrected and corrected distance visual acuities, monocular and binocular corrected near visual acuities, subjective refraction, biometry, intraocular pressure, and slit-lamp examination results. Surgery-related data included IOL power, IOL power calculation formula, A-constant value, and expected postoperative refraction.

During the postoperative visit the patients underwent a detailed ophthalmologic examination including monocular and binocular, uncorrected and corrected distance, intermediate and near visual acuities, subjective refraction (sphere, cylinder and spherical equivalent), slit-lamp examination of the anterior and posterior segment (status of the lid, conjunctiva, cornea, anterior chamber, lens and anterior vitreous of the eye, tilt and decentration), optical biometry (IOLMaster 500 or 700, Carl Zeiss Meditec AG, Jena, Germany), intraocular pressure. Adverse events, including posterior capsule opacification and Nd:YAG rates, were recorded during the follow-up period.

#### Patient-reported outcomes

At the postoperative visit patients were asked to complete a self-administered questionnaire which assessed the nature and impact of any photic phenomena, and a questionnaire that assessed quality of life and ability to function. The questionnaires have not been validated, but the questions were adapted from the validated PROWL questionnaire (Patient-Reported Outcomes with LASIK) [21]. The questions asked in the questionnaires can be found in Table 3 and Fig. 3.

After completion of the postoperative visit, the safety index was calculated as the ratio of the postoperative to the preoperative CDVA, and the efficacy index calculated as the ratio of postoperative uncorrected distance visual acuity (UDVA) to the preoperative CDVA. All individual visual acuity values were converted to decimal units before calculating the indexes.

#### Statistical analysis

A non-inferiority analysis was carried out on the assumption that there would be no inferiority between pre- and post-surgery values for monocular and binocular CDVA. The sample size was calculated based on an expected difference between the pre- and postoperative follow-ups of at least 0.1 logMAR, a power of 80% and a significance level of 0.025. The minimal recommended number of bilaterally implanted patients was 30. Taking into account possible screening failures, 36 patients were planned to be recruited.

Quantitative variables are presented as the number of non-missing observations (n), the arithmetic mean ± the standard deviation (SD), the minimum (min), and the maximum (max). For categorical variables, the number (n) and percentage (%) of subjects per category are presented. In addition to the standard summary statistics, the mean CDVA differences between the preoperative visit and the 4 to 12-month follow-up visit was analysed in a confirmatory manner using a one-sided paired t-test on a 2.5% significance level. All descriptive *p*-values were calculated on the assumption of a t-distribution. Statistical analysis was performed using SAS Version 9.4 or higher (SAS Institute Inc., Cary, USA).

## Results

### Patient characteristics

A total of 36 Caucasian patients (72 eyes) were included in the study. No patient had any sign of cataract, pre-cataract or any concomitant ocular disease. IOL power was calculated using the SRK/T formula in 44 eyes (60.1%), the Haigis formula in 20 eyes (27.8%) and the Holladay formula in 6 eyes (8.3%).

The preoperative characteristics are given in Table 1.

**Table 1:** Preoperative patient characteristics

	**n**	**Mean ± SD**	**Range**
**Patient characteristics**			
Age (years)	36	57.4 ± 6.0	47; 72
Gender (%, men / women)	36	52.8 /47.2	
Anterior chamber depth (mm)	71	3.25 ± 0.39	2.45; 4.17
IOL power (D)	72	21.9 ± 3.5	11.5; 27.0
Expected spherical equivalent (D)	68	-0.10 ± 0.21	-0.61; 0.33
**Refraction (D)**			
Sphere	72	0.82 ± 2.20	-7.0; 4.25
Cylinder	72	-0.53 ± 0.45	-1.75; 0
Spherical equivalent	72	0.67 ± 2.28	-7.0; 4.25
**Visual acuity (logMAR)**			
Monocular UDVA	69	0.46 ± 0.42	0.00; 1.30
Binocular UDVA	24	0.31 ± 0.34	0.00; 1.00
Monocular CDVA	72	0.01 ± 0.03	0.00; 0.20
Binocular CDVA	36	0.01 ± 0.04	0.00; 0.10
Monocular CNVA	53	0.01 ± 0.04	0.00; 0.20
Binocular CNVA	28	0.03 ± 0.05	0.00; 0.10

*CDVA* Corrected distance visual acuity, *CNVA* Corrected near visual acuity, *D* dioptre, *SD* Standard deviation, *UDVA* Uncorrected distance visual acuity

### Visual acuity

**Fig. 1:**
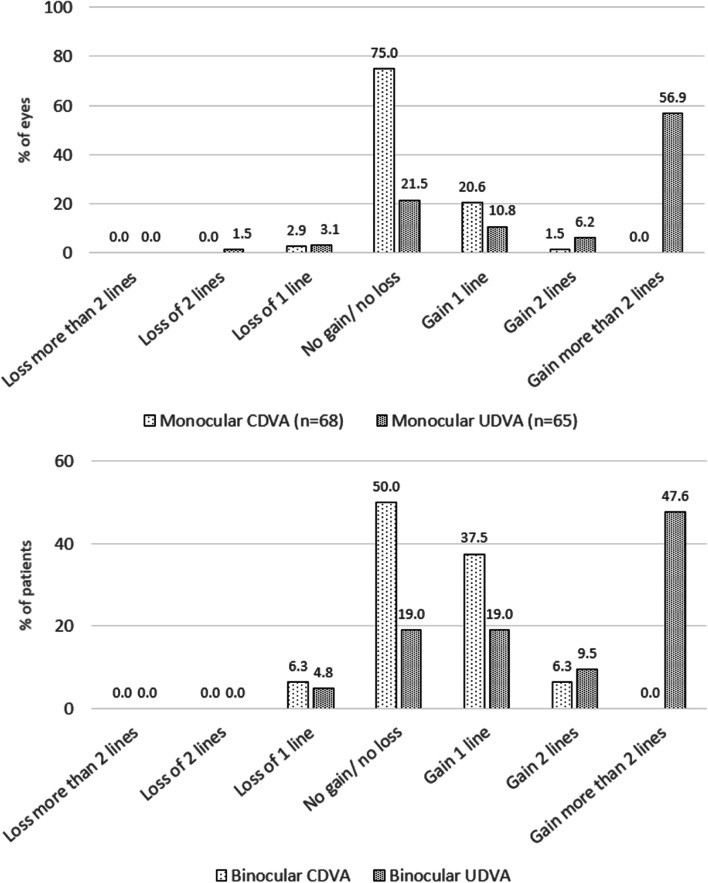
Change between preoperative and postoperative corrected and uncorrected, monocular and binocular visual acuity

Postoperative visual acuity outcomes are presented in Table 2. Gain or loss of EDTRS chart lines are presented in Fig. 1.

**Table 2:** Postoperative visual acuity outcomes (in logMAR)

	**n**	**Mean ± SD**	**Min; max**
**CDVA**			
Monocular	68	0.02 ± 0.04	0.00; 0.10
Binocular	32	0.07 ± 0.07	-0.10; 0.20
**UDVA**			
Monocular	68	0.04 ± 0.07	0.00; 0.30
Binocular	32	0.06 ± 0.07	0.00; 0.20
**UIVA**			
Monocular	68	0.14 ± 0.093	0.00; 0.40
Binocular	32	0.07 ± 0.06	0.00; 0.20
**DCIVA**			
Monocular	66	0.12 ± 0.09	0.00; 0.30
Binocular	31	0.07 ± 0.06	0.00; 0.20
**CIVA**			
Monocular	68	0.05 ± 0.07	-0.10; 0.20
Binocular	32	0.02 ± 0.08	-0.10; 0.20
**UNVA**			
Monocular	68	0.13 ± 0.8	0.00; 0.30
Binocular	32	0.08 ± 0.05	0.00; 0.20
**DCNVA**			
Monocular	66	0.12 ± 0.08	0.00; 0.30
Binocular	31	0.07 ± 0.05	0.00; 0.20
**CNVA**			
Monocular	68	0.04 ± 0.06	0.00; 0.20
Binocular	32	0.05 ± 0.05	0.00; 0.10

*CDVA* Corrected distance visual acuity, *UDVA* Uncorrected distance visual acuity, *UIVA* Uncorrected intermediate visual acuity, *DCIVA* Distance-corrected intermediate visual acuity, *CIVA* Corrected intermediate visual acuity, *UNVA* Uncorrected near visual acuity, *DCNVA* Distance-corrected near visual acuity, *CNVA* Corrected near visual acuity, *SD* Standard-deviation, *min* Minimum, *max* Maximum

Postoperatively, mean monocular CDVA was 0.02 ± 0.04 logMAR and mean binocular CDVA 0.07 ± 0.07 logMAR. The change from the preoperative baseline value was 0.01 ± 0.05 logMAR (*p* = 0.0478) for monocular measurements and 0.05 ± 0.07 logMAR (*p* = 0.002) for binocular measurements.

The difference between the mean pre- and post-surgery CDVA measurements (*p* = 0.0478 and *p* = 0.02) was not clinically significant, and the non-inferiority analysis confirms with a high degree of certainty that the two mean values pre- and post-surgery are similar (*p* < 0.0001).

All eyes had monocular CDVA better than 0.3 logMAR and 77.9% were better than 0.1 logMAR. The safety index and the efficacy index were 0.98 and 0.94 respectively.

### Refraction

**Fig. 2:**
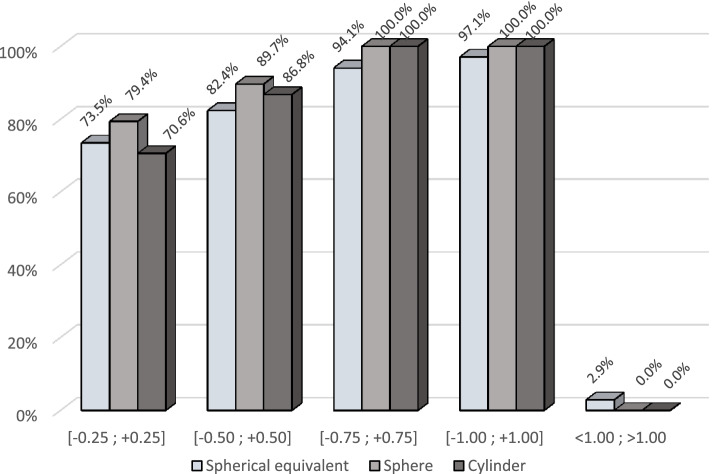
Refractive outcomes at 4 to 12 months post-surgery, by diopter class

Postoperatively, the mean refractive values showed statistically significant improvement compared with preoperative baseline. The mean postoperative sphere was -0.01 ± 0.30 D (*p* = 0.0009), the mean spherical equivalent -0.20 ± 0.35 D (*p* = 0.0064), and the mean cylinder -0.19 ± 0.29 D (*p* < 0.0001). The outcomes by dioptre class are shown in Fig. 2.

The accuracy, defined as the difference between the predicted and the postoperative spherical equivalent, was -0.10 ± 0.41 D, with 80.9% of the eyes within ± 0.5 D of the predicted value and 97.1% within ± 1.0 D.

### Patient-reported outcomes

In the postoperative questionnaire, the following percentages of patients reported spectacle independence 87.5% (for distance), 84.4% (for intermediate) and 78.1% (for near). The survey showed that 78.1% of the patients were completely / very satisfied with their spectacle-free vision. When corrective glasses were used, the rate of satisfaction reached 96.9% with one (3.1%) patient expressing complete dissatisfaction.

Preoperative vision satisfaction was compared with postoperative satisfaction and 90.6% of the patients declared being more satisfied with their uncorrected vision post-surgery than they were with their pre-surgery corrected vision. Two patients saw no difference and one patient preferred his corrected vision before the surgery. To the question “how happy or unhappy are you that you had refractive lens exchange surgery”, seven (21.9%) patients were completely happy, seventeen (53.1%) very happy, six (18.8%) somewhat happy, one (3.1%) somewhat unhappy and one (3.1%) completely unhappy. Twenty-seven (84.4%) patients declared that they would choose to have the surgery again, while five (15.7%) would decline surgery if they had a second chance.

**Fig. 3:**
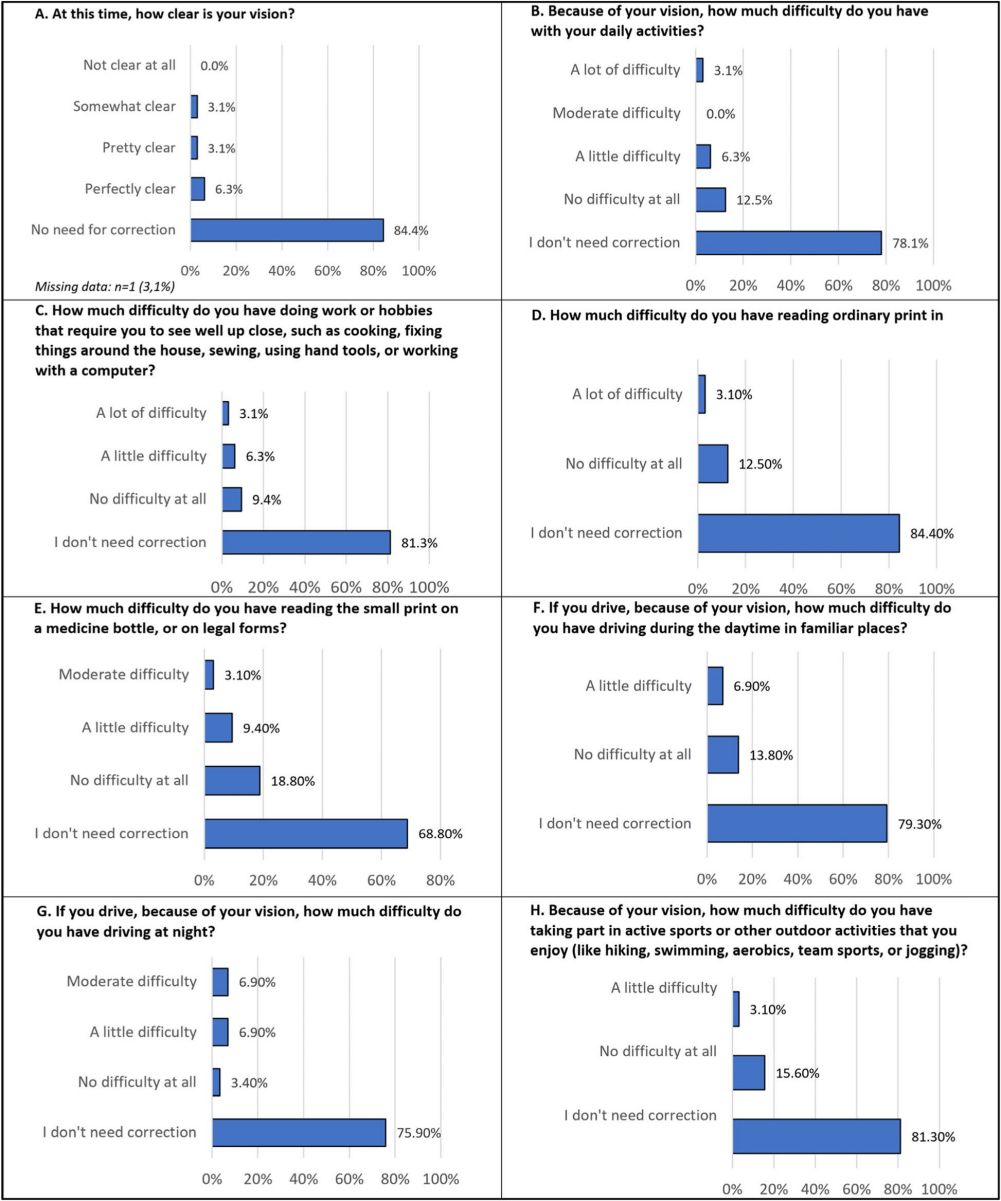
Results of the patient quality of life questionnaire (*n* = 32). Patients were asked to score their vision using their best correction. The full range of proposed answers for questions C to H was as follows: I don't need correction; no difficulty at all; a little difficulty; moderate difficulty; a lot of difficulty; I never try to do these activities because of vision; I never do these activities for other reasons. For the readability of the charts, the proposed options with no answers (0%) are not represented

**Fig. 4:**
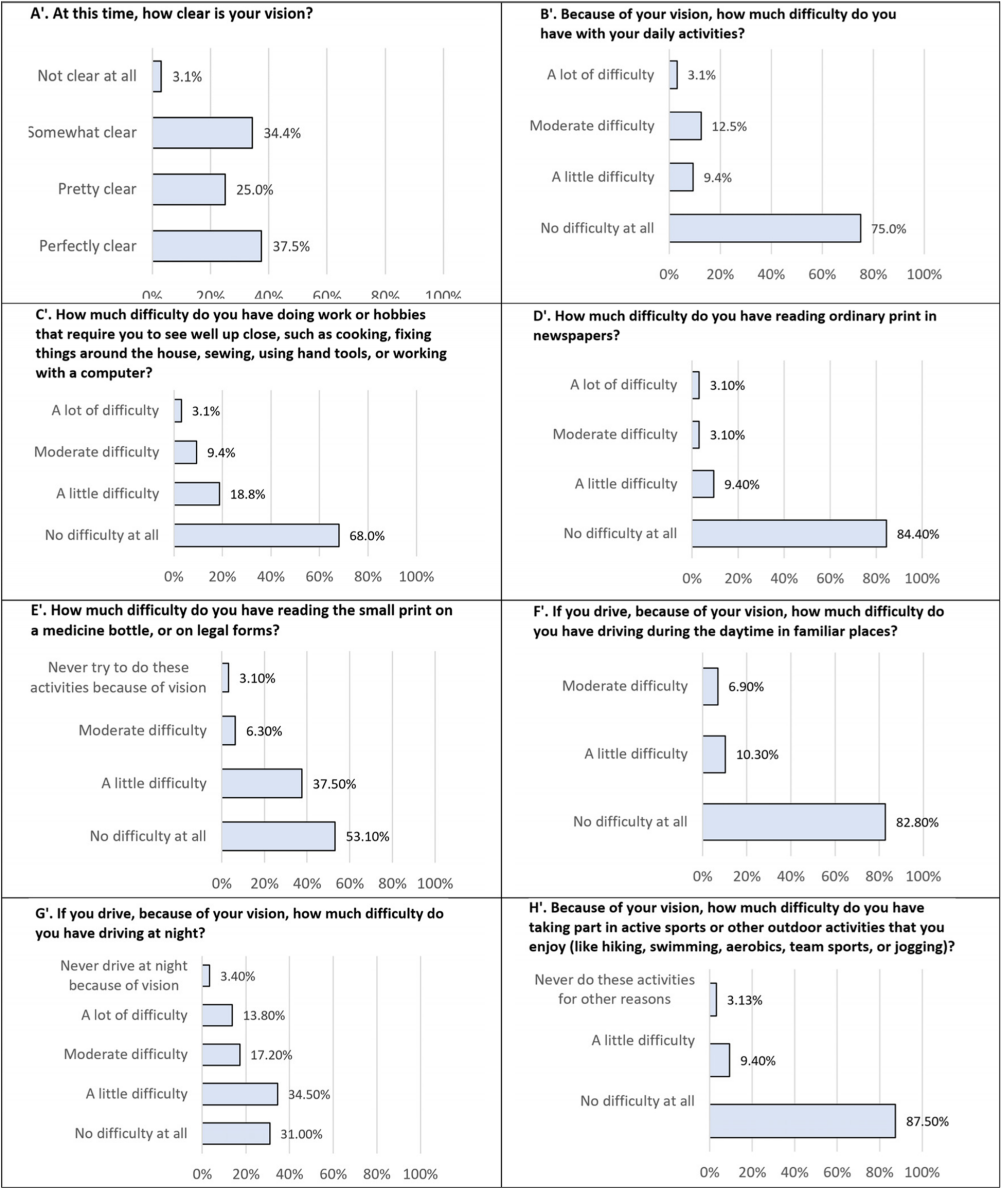
Results of the patient quality of life questionnaire (*n* = 32). Patients were asked to score their uncorrected vision. The full range of proposed answers for questions C’ to H’ was as follows: I don't need correction; no difficulty at all; a little difficulty; moderate difficulty; a lot of difficulty; I never try to do these activities because of vision; I never do these activities for other reasons. For the readability of the charts, the proposed options with no answers (0%) are not represented

Detailed results on patients’ quality of life are given in Figs. 3 and 4, and results on visual dysphotopsia are provided in Table 3.

**Table 3:** Patient experience questionnaire

In the last 7 days, how often have you seen?		When symptoms were experienced, how bothersome were they?	
**Double images**			
N	32	n	3
Never	29 (90.6%)	Extremely bothersome	0 (0.0%)
Rarely	0 (0.0%)	Very bothersome	0 (0.0%)
Sometimes	1 (3.1%)	Somewhat bothersome	3 (100%)
Often	1 (3.1%)	A little bothersome	0 (0.0%)
Always	1 (3.1%)	Not at all bothersome	0 (0.0%)
**Glare**			
N	32	n	14
Never	18 (56.3%)	Very	2 (14.3%)
Occasionally	4 (12.5%)	Quite	5 (35.7%)
Quite often	6 (18.8%)	A little	6 (42.9%)
Very often	4 (12.5%)	Not at all	1 (7.1%)
**Halos**			
N	32	n	30
Never	2 (6.3%)	Very	5 (16.7%)
Occasionally	10 (31.3%)	Quite	5 (16.7%)
Quite often	9 (28.1%)	A little	16 (53.3%)
Very often	11 (34.4%)	Not at all	4 (13.3%)
**Starbursts**			
N	32	n	24
Never	8 (25.0%)	Extremely bothersome	2 (8.3%)
Rarely	1 (3.1%)	Very bothersome	5 (20.8%)
Sometimes	5 (15.6%)	Somewhat bothersome	7 (29.2%)
Often	9 (28.1%)	A little bothersome	9 (37.5%)
Always	9 (28.1%)	Not at all bothersome	1 (4.2%)

### Safety

No cases of tilt were observed following implantation and there was only one (1.4%) case of decentration (downward by 0.3 mm).

Postoperatively mean intraocular pressure, status of the lid, conjunctiva, cornea, anterior chamber, lens and anterior vitreous of the eye were assessed during slit lamp examination. All observations were normal, except for two eyes (2.8%) with postoperative conjunctival hyperaemia and one eye (1.4%) with trace of vitreous flare/haze.

Patient related adverse events reported during the study included: eight eyes (11.1%) of five patients receiving a Nd:YAG laser treatment and one eye (1.4%) with vitreous detachment. The latter was mild and not related to the IOL implanted. No IOL dislocation or explantation occurred during the study.

## Discussion

In this study we evaluated the safety, efficacy and patient satisfaction after bilateral implantation of the trifocal AT LISA tri 839MP IOL in presbyopic eyes. We focused on patients with presbyopia as published data for this sub-group of patients is scarce.

Patients who opt for IOL implantation to treat presbyopia seek spectacle-independence and optimal vision at all distances. Although these patients seek an improvement in their near and intermediate vision, they do not expect that this will be at the expense of distance visual acuity.

Preoperatively, the patients had a very high level of CDVA that was satisfactorily maintained postoperatively. In our study the change from the preoperative baseline value for CDVA was 0.01 ± 0.05 logMAR (*p* = 0.0478) for monocular measurements and 0.05 ± 0.07 logMAR (*p* = 0.002) for binocular measurements. These differences were clinically non-relevant, demonstrating the safety of the AT LISA tri 839MP IOL in maintaining a high level of postoperative CDVA.

A change in VA of 0.3 logMAR (3 lines, or 15 letters) is regarded by the NEI and FDA as being the minimum level at which visual change can be deemed clinically significant [22, 23].

Mean visual acuity results were very good and uncorrected binocular vision was equal to or less than 0.08 logMAR for the three distances. Visual acuity outcomes were consistent with previous reports on IOL implantation in presbyopic patients: Mojzis et al. with the same IOL reported binocular values of 0.05 ± 0.08 logMAR, 0.08 ± 0.10 logMAR and 0.20 ± 0.12 logMAR for far, intermediate and near vision respectively, 3 months after implantation [17]. Fernández-García et al. reported binocular values of 0.01 ± 0.02 logMAR, 0.02 ± 0.06 logMAR and 0.04 ± 0.05 logMAR for far, intermediate and near vision respectively, 3 months after implantation of the FineVision trifocal lens [24]. Nicula et al. reported values better than 0.00 logMAR for all three distances 1 year after implantation of the AcrySof PanOptix (Alcon) [25].

A prerequisite for good visual acuity is an accurate prediction and correction of the refraction errors. The accuracy between the predicted and the postoperative spherical equivalent was -0.10 ± 0.41 D, with 80.9% of the eyes within ± 0.5 D and 97.1% within ± 1.0 D of the predicted value. These results were consistent with the data recently published with the Versario 3F (Valeant Med Sp.z o.o, Warsaw, Poland) or the PanOptix (Alcon Inc., Fort Worth, USA) multifocal IOLs with, respectively, 72% and 82% of the eyes within ± 0.5 D of planned correction [25, 26].

Five patients lost one line of corrected or uncorrected distance VA following the surgery, and one lost two lines. In three patients, the loss could be attributed to posterior capsule opacification (PCO), which was clinically significant in two cases. No explanation for the loss of lines could be found for the remaining three patients. One of these patients was “somewhat” dissatisfied with their surgery, but the other two were very satisfied with the outcome and were spectacle-independent despite the loss of a line of vision. As we have noted above, a difference of less than 0.3 logMAR is not considered to be clinically significant, providing a possible explanation as to why the loss of a line of vision did not lead to dissatisfaction.

Results from the patient questionnaire highlighted the fact that presbyopic patients represent a patient group with high postoperative visual expectations. Overall patient satisfaction was very high, with 93.8% of the patients expressing satisfaction with the surgery outcomes, and 78.1% expressing complete satisfaction with their uncorrected vision following the surgery.

Questions related to near and intermediate vision showed successful restoration of visual function for those distances with more than 93% of patients stating that they had little / no difficulties in reading normal or small prints without glasses, and 87.5% stating that they had little / no difficulties in doing activities necessitating intermediate distance visual functions.

Postoperative dysphotopsia can remain a cause of patient dissatisfaction after implantation of a multifocal IOL [27]. In our study, halos and starbursts were the two most frequently reported symptoms, followed by glare.

Other published reports suggest that dysphotopsias experienced by some patients following surgery are important factors that could limit patient satisfaction [28–30]. Our study found that patients were very satisfied with their daytime distance vision (96.9% no / little difficulty in carrying out normal outdoor daytime activities even without glasses, 93.1% no / little difficulty for driving at daytime) but satisfaction with mesopic vision was reduced with 65.5% of the patients expressing little / no difficulty for driving at night without glasses. The reduction of visual function in dim conditions is well documented following implantation of multifocal IOLs [31] and is thought to be the result of decreased contrast sensitivity [32, 33] and an increased impact of visual symptoms such as halos, glare and starbursts [34, 35].

Five patients said that they would not wish to undertake the surgery again. Three of these patients reported at least one severe dysphotopsia: starbursts were the most commonly cited. A fourth patient reported mild starbursts. These four patients also reported moderate or severe halos and glare. These translated into difficulties with night driving, severe for two patients [#1,2], and moderate or mild difficulties with close work and reading small print [#1–4].

Surprisingly, the fifth patient [#5] reported no dysphotopias and no impact on daily functioning, and reported that she is very happy [with her vision], very satisfied and completely spectacle independent, but would still refuse surgery if it were offered again. Another patient [#2], who reported severe halo and persistent starburst, maintained that he was very happy with his current vision, and was more satisfied with his current vision than his uncorrected pre-surgery vision, but would still not undertake surgery a second time. Another of the patients [#3] who reported severe visual effects, nonetheless claimed to be very satisfied with his vision, and to have an improved quality of life, but would still refuse surgery. Patient [#1] stated that his quality of life had not been affected but would not undergo surgery again. Only one patient [#4] stated that his quality of life was worse, and was dissatisfied with the outcome.

These visual symptoms are relatively common and have been reported by many authors, even if their frequency and severity vary significantly between studies [9, 36–38] probably due to factors such as the length of time following the surgery [17, 39, 40], the patient visual acuity level before the surgery [41], or the heterogeneity of the methods employed.

This study specifically addressed the safety and performance of the AT LISA tri 839MP IOL in the treatment of presbyopia. The patient population undergoing presbyopic lens exchange is younger than the cataract population and, in our opinion, additional long-term studies are necessary to further determine the long-term benefit-risk balance. One of the shortcomings of this study is its single follow-up visit. Neuroadaptation is important in the final visual perception and satisfaction following multifocal IOL implantation. In cataract patients, brain adaptation can take up to 6 months [42, 43]. Patients participating in this study were examined between 4 and 12 months post-surgery and therefore the neuroadaptation process may not have had sufficient time to fully stabilise. As visual acuity and refraction have been demonstrated to be stable from 1 month after surgery [14, 44], this limitation was however, not expected to significantly affect the main outcomes of this study.

Due to the retrospective nature of the initial part of the study (collection of baseline data), there are some gaps in the data for Spherical Equivalent (SE), Anterior chamber Depth (ACD) and UDVA. However, for SE, only 5.4% of data were missing and for ACD and UDVA, < 5% of the data were missing. Statistically, this will have no relevant impact on observations made for these values. Regarding the primary endpoints, data for all patients was available.

Another drawback is that the study was partially retrospective and therefore carries some risks of selection bias and of non-standardisation of the preoperative data. Ideally we would have been able to compare pre- and post-operative UIVA, UNVA and CDVA, but these measurements were not available for all patients. These risks were however, limited by enrolling participants according to objective inclusion and exclusion criteria defined in the protocol and by using externally monitored enrolment logs.

Another potential limitation is that patients were enrolled with a minimum preoperative CDVA of 0.2 logMAR, which is lower than in some other studies. However, patients were inspected with a slit-lamp at entry for signs of cataract, and none were observed.

## Conclusions

In conclusion, the results of our study showed that the AT LISA tri 839MP IOL is safe and effective in a presbyopic population. Refraction was accurately corrected and spectacle-free vision at all distances restored for most patients. Presbyopic patients are a population who opt for surgical correction through a strong desire to improve their quality of life. It is therefore of utmost importance that it is clearly explained to patients what can reasonably be expected from the surgery, including its potential limitations, specifically with regards to contrast sensitivity and night dysphotopsia. Patients whose visual expectations have been met can however achieve a high level of satisfaction.

## Data Availability

The datasets used and/or analyzed during the current study are available from the corresponding author on reasonable request.

## Abbreviations

ACD: Anterior chamber Depth
CDVA: Corrected distance visual acuity
CIVA: Corrected intermediate visual acuity
CNVA: Corrected near visual acuity
D: Dioptre
DCIVA: Distance-corrected intermediate visual acuity
IOL: Intraocular lens
logMAR: Logarithm of the minimum angle of resolution
NdYAG: Neodymium-doped yttrium aluminium garnet
Min: Minimum
Max: Maximum
SD: Standard deviation
SE: Spherical Equivalent
UDVA: Uncorrected distance visual acuity
UNVA: Uncorrected near visual acuity
